# Nucleases as molecular targets for cancer diagnosis

**DOI:** 10.1186/s40364-021-00342-4

**Published:** 2021-11-22

**Authors:** Alien Balian, Frank J. Hernandez

**Affiliations:** 1 Department of Physics, Chemistry and Biology, Linköping University, 58185 Linköping, Sweden; 2 Wallenberg Centre for Molecular Medicine, Linköping University, Linköping, Sweden

**Keywords:** Nuclease, Nuclease activity, cancer biomarkers, cancer diagnostics, Nucleic acid probes, novel diagnostics

## Abstract

Early cancer diagnosis is a crucial element to improved treatment options and survival. Great research efforts have been made in the search for better performing cancer diagnostic biomarkers. However, the quest continues as novel biomarkers with high accuracy for an early diagnosis remain an unmet clinical need. Nucleases, which are enzymes capable of cleaving nucleic acids, have been long considered as potential cancer biomarkers. The implications of nucleases are key for biological functions, their presence in different cellular counterparts and catalytic activity led the enthusiasm towards investigating the role of nucleases as promising cancer biomarkers. However, the most essential feature of these proteins, which is their enzymatic activity, has not been fully exploited. This review discusses nucleases interrogated as cancer biomarkers, providing a glimpse of their physiological roles. Moreover, it highlights the potential of harnessing the enzymatic activity of cancer-associated nucleases as a novel diagnostic biomarker using nucleic acid probes as substrates.

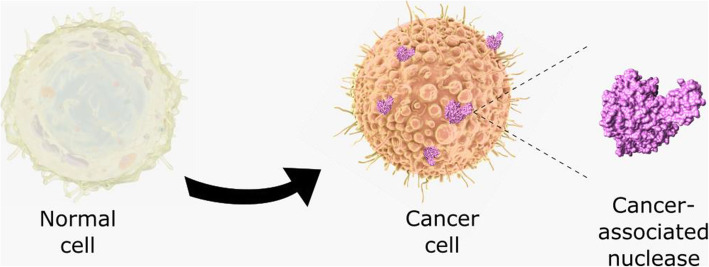

## Introduction

Despite great progress in therapeutic and diagnostic fields, cancer is the second leading cause of death globally [1]. The mortality is mainly due to failure in early detection [2]. It has been established that early cancer diagnosis has a pivotal impact on treatment response for curative intent [3], and its relevance has been emphasized in all cancer types [4–8].

Currently available diagnostic strategies can be divided into two main categories; medical imaging and biomarker analysis, along with a clinical examination to establish a clinical picture. The gold standard method to confirm a cancer diagnosis involves an invasive procedure of obtaining a biopsy [9].

However, lack of sensitivity and/or specificity remains an issue in imaging modalities [9–12]. Moreover, concerning biomarkers, a plethora of promising biomolecules have been suggested for early detection [2]. However, a small proportion has been approved for use in clinical routine [13, 3]. Furthermore, no biomarker appears to provide sufficient diagnostic power in the clinic when used alone [2].

As such, there is an increasing need to identify biomarkers for early detection [3]. Besides, platforms with a wide dynamic range capable of capturing subtle biomolecular alterations at as a low scale as picomolar and femtomolar levels are also a demand [2, 3, 10, 14].

Among the biomolecules that garnered research interest as promising cancer biomarkers are nucleases. From a molecular standpoint, nucleases are a group of enzymes that degrade nucleic acids by hydrolyzing the phosphodiester bonds between the ribose moieties. These enzymes can be widely classified, according to the substrate preference, into DNases and RNases that catalyze the cleavage of DNA and RNA, respectively [15].

Investigating alterations in nucleases in correlation with cancer can be motivated by the involvement of these enzymes in a variety of essential functions. For instance, nucleases contribute to apoptosis [16], innate immunity against bacterial and viral pathogens [17]. Not less importantly, they are key players in DNA replication and translation, acting as guardians of the genetic content [18–23]. Hence, it is not surprising that several studies have interrogated the potential role of nucleases as cancer biomarkers.

In this review, we present nucleases that have been reported as promising biomarkers in several cancer types. Additionally, we shed light on the utility of cancer-associated nuclease activity as a novel cancer biomarker, that holds promise for the translation into a noninvasive diagnostic methodology in clinical practice.

### Nucleases in cancer

**Fig. 1:**
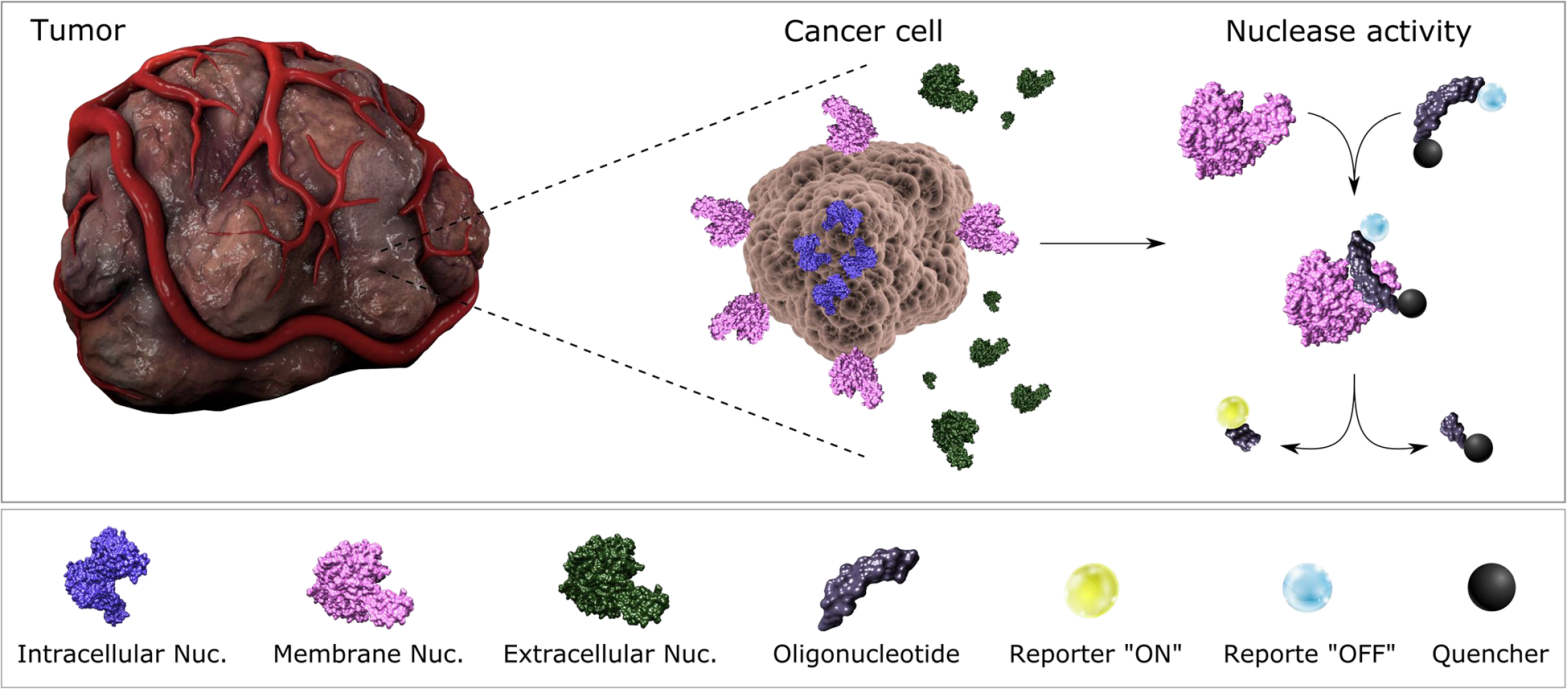
Cancer-associated nucleases and nuclease activity: nucleases are expressed intracellularly, extracellularly, or on the cell membrane in cancer cells. The catalytic activity of cancer nucleases could be harnessed in cancer detection using activatable nucleic acid probes as substrates

Nucleases are expressed and enzymatically active intracellularly and extracellularly [15]. This abundant availability confounded with their catalytic enzymatic activity renders nucleases into diagnostic targets that can be exploited on a molecular but also functional level, by the incorporation of nucleases in the appropriate detection system. Most of the studies, as shown below, focus on the investigation of alteration in nuclease expression at protein and/or RNA level in various cellular counterparts, using techniques such as immunohistochemistry (IHC), enzyme-linked immunosorbent assay (ELISA), and polymerase chain reaction (PCR). Nonetheless, not much has been reported regarding harnessing the catalytic activity of nucleases in cancer diagnosis, due to the lack of tools and methods for their specific detection. Although, promising results from recent research might imply a change in this regard. Figure 1 provides a schematic representation of intracellular, cell membrane, and extracellular nucleases in cancer, emphasizing the possibility of targeting the catalytic function of these enzymes as a biomarker using nucleic activatable acid probes as substrates.

**Fig. 2:**
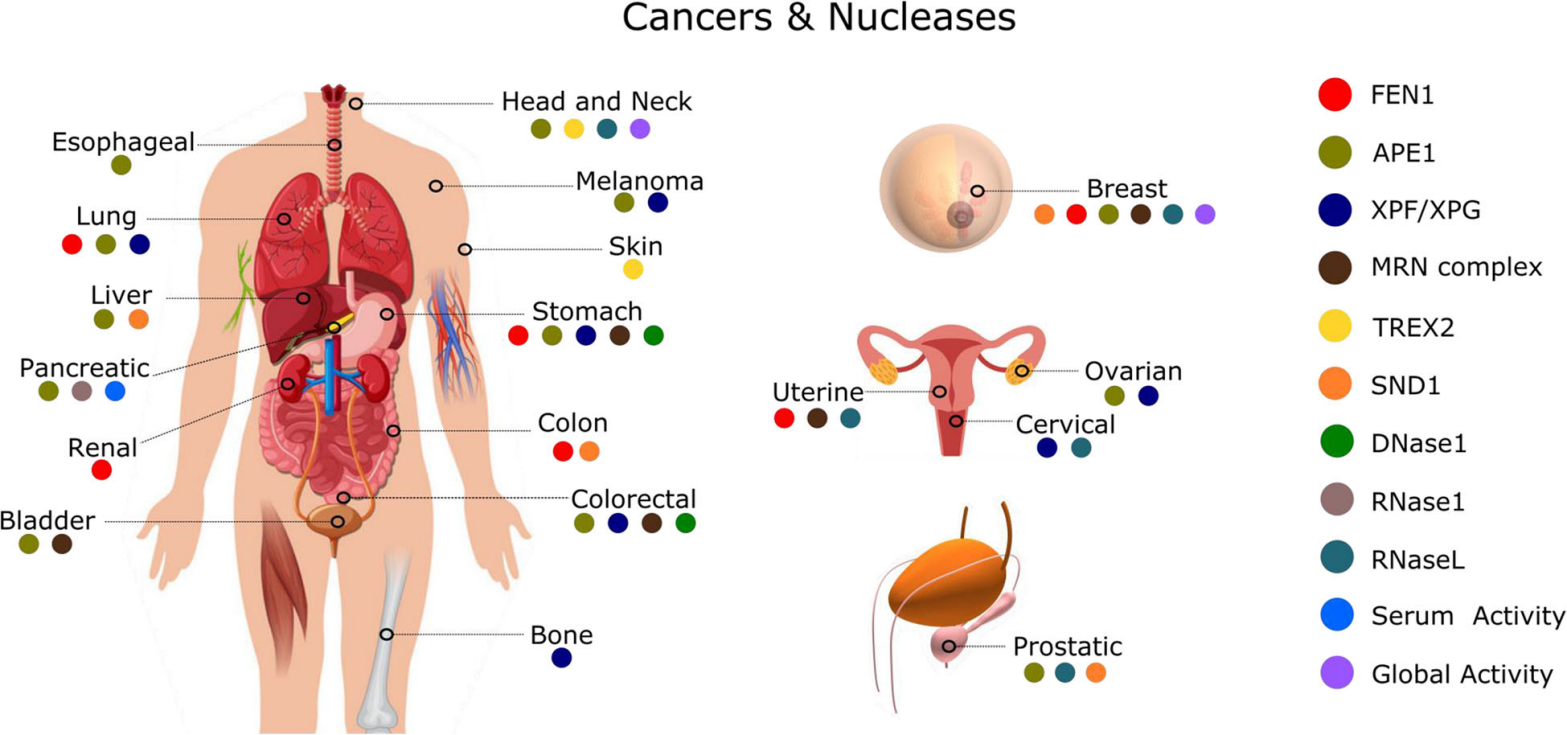
Nucleases and cancer types: Each nuclease, resembled by a specific color, is linked to the respective cancer type(s) for which it is reported. The cancer types are represented and indicated in the human body outline

Beyond being DNases, RNases, or sugar non-specific, nucleases can be classified into several categories based on the features of their catalytic activity. For instance, endonucleases cleave within nucleic acid while exonucleases cleave at the 5′-end or 3′-end. Nuclease activity can be metal ion-dependent or independent. Nucleases can also exhibit cleavage preference towards single-stranded or double-stranded nucleic acids. Some nucleases are structure-specific or sequence-specific [15]. In this review, we have depicted cancer related nucleases by the organ where the nuclease was described as a biomarker. In Fig. 2, each nuclease was assigned with a specific color which is placed in the organ of the human body, where nucleases are respectively involved in cancer diagnosis.

Globally, this section focuses on nucleases that have been frequently reported as potential biomarkers in correlation with several cancer types, along with their physiological roles (Table 1).

**Table 1:** Nucleases in correlation with cancer types

Name and type of nuclease	Type of cancer	Nuclease alteration	References
FEN1 DNase/RNase, endonuclease, exonuclease	uterine, colon, lung, and kidney cancer	Over expression of mRNA	[24]
	breast, gastric	Overexpression of mRNA and protein	[24, 25]
APE1 DNase, endonuclease	non–small cell lung-cancer	High expression at protein level, difference in subcellular expression patterns between cancer and benign	[26, 27]
	lung cancer	lower enzymatic activity correlated with higher risk	[20]
	ovarian	staining limited to the nuclei in healthy tissues, both nuclear and cytoplasmic expression was detected in cancer tissues	[28]
	gastric	Nuclear staining more prevalent than cytoplasmic staining in cancer tissues Elevated serum protein associated with lymph node metastasis	[29, 30]
	gastro-esophageal	overexpression	[29]
	breast	overexpression Increased cancer susceptibility correlated with the SNP variant Asp148Glu	[29, 31, 32]
	pancreatico-biliary	Overexpression, negative cytoplasmic expression with nuclear APE1 expression correlated with poor tumor differentiation, greater stage and vascular invasion	[29]
	prostate cancer and prostatic intraepithelial neoplasia	Increased cytoplasmic and nuclear expression	[33]
	human hepatocellular carcinoma	upregulation, elevated serum levels	[34, 35]
	bladder	Elevated serum concentration	[36]
	colorectal	stem cells exhibit higher expression compared with non-stem cells Asp148Glu and hOGG1 Ser326Cys polymorphisms associated with increased risk, higher frequency of the polymorphisms detected in blood samples from CRC patients compared with healthy subjects	[37, 38]
	head and neck	correlation between loss of nuclear expression and better prognosis and treatment response	[39]
	melanoma	high expression at mRNA level correlated with poor survival	[40]
XPF/XPG DNase, endonuclease	Lung, cervical and ovarian	ERCC1 overexpression associated with poor response to platinum-based chemotherapy Poor overall survival in ovarian cancer associated with high expression of XPG at RNA level	[21, 41]
	non-small cell lung cancer	Improved treatment response correlated with negative staining of tumor sample	[42]
	melanoma	ERCC1 deficiency correlated with improved response to cisplatin therapy	[43]
	gastric	improved treatment response and survival in correlation with elevated ERCC1 protein elevated expression of XPG	[21, 44]
	colorectal	improved survival in correlation with low ERCC1-mRNA expression in the tumors	[45]
	osteosarcoma	improved treatment response to a platinum-based therapy in XPF and XPG knocked down cell line	[46]
MRN Complex DNase, endonuclease/exonuclease	breast	loss of expression in breast cancer tissues	[47]
	Colorectal	mutations in MRE11 resulting in reduced expression of MRE11 and impaired function of the MRN complex	[48]
	endometrial	loss of MRE11 expression, loss of MRE11 correlated with the loss of the other components of the complex, mutations in *MRE11* resulting in reduced expression of MRE11 and impaired function of MRN complex	[48, 49]
	bladder	High expression of MRE11 correlated with improved survival	[50]
	gastric carcinoma with high level microsatellite instability	mutations of the intronic poly(T)11 repeat in MRE11	[51]
TREX2 DNase, exonuclease	skin carcinogenesis	Knock out mouse model	[52]
	SCC	deregulated expression	
	HNSCC	deregulated expression, decreased relative dsDNase activity in the R156L variant	
SND1 RNase, endonuclease	human hepatocellular carcinoma	overexpression increased angiogenesis	[53, 54]
	breast cancer	mRNA overexpression correlated with reduced survival	[55]
	prostate cancer	overexpression of protein and mRNA, positive correlation with tumor grade	[56]
	colon cancer	Overexpression of mRNA	[57]
DNaseI endonuclease	gastric carcinoma, colorectal carcinoma	high frequency of DNaseI phenotype2	[58, 59]
RNaseL endonuclease	prostate cancer	RNaseL mutations, SNPs	[60, 61]
		decreased enzymatic activity of the variant	[62]
	uterine cervix, HNSCC and breast cancer	a correlation between increased cancer risk and RNaseL SNP rs3738579	[63]
RNaseI endonuclease	Pancreatic adenocarcinoma	difference in glycosylation between healthy and cancer	[64, 65]
Serum RNase activity	pancreatic carcinoma	elevated serum ribonuclease activity	[66]

Table 1 summarizes literature findings about nucleases involved in cancer and alteration of nucleases in correlation with respective cancer types

Moreover, in this review, we provide a complete literature summary of the analytical methods used for the detection of nuclease or nuclease activity for the nucleases discussed in this section (Table 2).

**Table 2:** Nuclease detection methods

Nuclease	Detection method	Reference
FEN1	Cancer Profiling Array I	[24]
	IHC	[24, 25]
	semiquantitative reverse transcription-PCR	[25]
APE1	IHC	[26–29, 33, 39]
	radioactivity-based or fluorescence- based nuclease activity assay	[20]
	ELISA	[30, 34, 36]
	liquid chromatography and tandem mass spectrometry with isotope dilution	[31]
	genotyping assays and in silico prediction	[32]
	RT-qPCR	[35]
	qPCR	[37]
	PCR	[38]
	RFLP	[38]
	whole genome gene expression of melanoma tumors, using Illumina DASL approach	[40]
XPF/XPG	IHC	[21, 42, 44]
	RT-qPCR	[45]
MRN Complex	IHC	[47–50]
	PCR	[48, 49, 51]
TREX2	knock out mouse model, IHC, ssDNA and dsDNA degradation assays	[52]
SND1	IHC	[53, 56]
	chicken chorioallantoic membrane assay, human umbilical vein endothelial cell differentiation assay	[54]
	RT-qPCR	[57]
DNaseI	phenotyping conducted on urine samples from participants, using electrophoresis in thin polyacrylamide gel followed by immunoblotting with an antihuman DNaseI antibody	[58, 59]
RNaseL	gene sequencing, 5′ nuclease TaqMan® allelic discrimination assay, genotyping using PCR and WAVE DHPLC	[60, 61]
	enzymatic assay using rRNA as a substrate	[62]
	analysis of tumor DNA and genotyping of somatic tissues of patients	[63]
RNaseI	WB, ELISA, and immunoprecipitation	[64, 65]
Serum RNase activity	serum RNase enzymatic activity was assayed using two substrates: t-RNA (T) from *E. coli* MRE 600 and the synthetic polycytidylic acid (poly-C). Elevated serum ribonuclease activity (SRA) was expressed in terms of the amount of bovine serum RNaseA from bovine pancreas in ngeq/ml that yields the same extinction Coeff. at the 260 nm wavelength.	[66]

Considering this wide diversity of nucleases and being more than one nuclease reported for each cancer type and using different analytical methods for detection of the same nuclease in one or more cancer types, this section describes the nucleases that have been reported as biomarkers in cancer one by one.

### FEN1

Flap endonuclease1 (FEN1) is a multifunctional enzyme that exerts 3 different activities: gap-dependent endonuclease, exonuclease, and 5′-flap endonuclease with the latter being dominant. Hence, FEN1 can be identified as a key regulator of genome stability due to its role in DNA repair through the removal of 5′-flaps during long-patch base excision repair (BER) and DNA replication through involvement in the maturation of Okazaki fragments [19]. According to the Cancer Genome Atlas (TCGA) database, overexpression of FEN1 has been associated with several types of cancers including gastric, breast, prostate, lung, pancreatic, and brain cancer. Data regarding breast cancer showed a correlation between increased FEN1expression and level of malignancy [19].

This finding is consistent with results from a comprehensive evaluation of FEN1 expression in various human tumors that has been conducted using Cancer Profiling Array I, which includes normalized cDNAs from paired samples tumors and corresponding normal tissues. Higher expression of FEN1-mRNA was found in a large number of tumors including breast, uterine, stomach, colon, lung, and kidney cancer compared with matched normal tissues. Among all types, the greatest expression of FEN1-mRNA has been found in breast cancer samples. Similarly, IHC analysis of FEN1 protein expression in breast cancer samples revealed high expression in these samples, and a positive correlation between expression and increased relative cancer risk was found. Hypomethylation of the cytosine-phosphate-guanine (CpG) islands within the FEN1 promoter was demonstrated as the underlying mechanism for the aberrant expression of FEN1 in tumors. Hence, FEN1 overexpression and FEN1 promoter hypomethylation were suggested to be promising biomarkers in cancer [24]. Additionally, the role of FEN1 as a diagnostic biomarker has been suggested by Wang and Xie. In their study, analysis of FEN1-mRNA and protein expression using semiquantitative reverse transcription-PCR and IHC in paired samples of gastric cancer and corresponding normal tissues demonstrated FEN1 overexpression in gastric cancer. High expression of FEN1 at the protein level was positively correlated with tumor size and TNM stage [25].

He et al. have demonstrated, applying both Western Blot (WB) and IHC staining on breast cancer tumor specimens, that FEN1 is overexpressed in breast cancer compared with the adjacent healthy tissue derived from the same patients. Using WB, FEN1 overexpression has been demonstrated in breast cancer cell lines MDA-MB-231, MCF7, and MDA-MB-435 when compared with the healthy breast cell line MCF10A [19]. Suppression of FEN1 in the cell line MCF7 led to reduced cell growth and foci formation. Reversely, when FEN1 was overexpressed in MCF10A, that have low endogenous FEN1 expression, an enhancement in cell growth and induced foci formation was observed. By reproducing this result in other breast cancer cell lines; MDA-MB-231, T47D, and HCC1937 as well as lung and colorectal cancer cell lines, it has been concluded that FEN1 promotes cancer cell growth [19]. Furthermore, analysis of FEN1 expression in large cohorts of breast and ovarian cancer has correlated FEN1 overexpression at mRNA and protein level with poor prognosis and treatment response to chemotherapy and endocrine therapy, suggesting the potential of FEN1 as a prognostic and predictive biomarker in these types of cancer [67].

Furthermore, the correlation between deficient DNA repair and carcinogenesis is well established where the loss of DNA repair contributes to genomic instability and thereby carcinogenesis [21]. Due to its pivotal role in DNA repair, dysregulated FEN1 results in deficient DNA repair and subsequent accumulated mutations and predisposition to cancer, as evident in preclinical studies [21]. For instance, null mutation of FEN1 in mouse model harboring heterozygous mutation resulted in cancer predisposition including lymphoma and contributed to gastrointestinal cancer when combined with heterozygous mutation in adenomatous polyposis coli (APC) gene [68].

### APE1

Human apurinic/apyrimidinic endonuclease1 (APE1) is a multifunctional protein that plays a crucial role in long-patch and short-patch BER. Particularly, it is the major responsible endonuclease for identification and cleavage of cytotoxic apurinic/apyrimidinic (AP) sites which arise spontaneously or in response to DNA damaging irradiation. If not processed, AP sites exhibit cytotoxicity mainly attributed to interfering with DNA forks [21]. DNA oxidative damage repaired by APE1 can arise due to smoking, exposure to heavy metals but also due to an internal process of inflammation [20].

APE1 is expressed in the nuclei and/or cytoplasm of cancer cells, and a multitude of studies have been conducted to investigate the role of APE1 as a prognostic and predictive biomarker in cancer, with the expression status indicating even a diagnostic potential [69]. In an IHC analysis of 103 tumor tissues obtained from non–small cell lung cancer patients, a proportion as high as 73.8% showed high expression of APE1 protein, with differences in subcellular localization between cancer and normal samples. While APE1 was only present in the nucleus of normal cells, staining showed APE1 expression in both the nuclei and cytoplasm of cancer cells [26]. Similar patterns of subcellular distribution were found in non-cancerous regions in tumor specimens that exhibited nuclear staining and cancer cells that showed both nuclear and cytoplasmic expression of APE1 [27]. Interestingly, the enzymatic activity of APE1 has been exploited to assess lung cancer susceptibility. The enzymatic activity of the nuclease has been measured in protein extracts from peripheral blood mononuclear cells (PBMC) using a radioactivity-based or fluorescence-based assay. A 30-mer of synthetic DNA duplex labeled with Phosphorus-32 (^32^P) or Yakima yellow at the 3′ end was used as an APE1 substrate in the radioactivity and the fluorescence-based reaction, respectively. Since the substrate contains a synthetic AP-site, an abasic furanyl-site, it allows measuring APE1 incision activity at this site that cleaves the 30-mer into 15-mer oligonucleotide labeled reaction products, that are further quantified. Samples from lung cancer patients showed a significantly lower enzymatic activity compared with the ones obtained from the healthy subjects. Therefore, it was suggested that inter-individual differences in APE1 activity in PBMC are associated with lung cancer susceptibility where lower activity indicates higher risk. Furthermore, APE1 Asp148Glu polymorphic variant which has gathered disagreement on being associated or not with lung cancer susceptibility has not been correlated with APE1 enzymatic activity level nor with lung cancer risk according to this study [20]. In agreement with findings by Wang, D et al. [26], IHC analysis showed similar expression patterns when comparing normal and cancerous ovarian tissues. While APE1 staining was limited to the nuclei, both nuclear and cytoplasmic expression was detected in cancer tissues [28]. Nonetheless, nuclear APE1 expression visualized by IHC was more prevalent than the cytoplasmic expression in ovarian cancer according to another study [29].

In a screening of breast tissues of normal and invasive cancer samples, IHC staining revealed similar patterns of high nuclear expression of APE1 in both tumor and normal tissue samples [70]. On the contrary, a high expression level of APE1 has been detected in breast cancer tissues compared with normal counterparts using a different approach. In this study, APE1 levels were measured using liquid chromatography and tandem mass spectrometry with isotope dilution. This technique eliminates antibody-related measurement bias and offers a more accurate, and quantitative assessment of APE1 levels compared with the conventionally used quantitative real-time PCR and WB [31]. Furthermore, genotyping assays and in silico prediction have shown involvement of aspartic to glutamic acid at codon 148 (Asp148Glu) single nucleotide polymorphism (SNP) variant of APE1 in breast cancer progression, indicating that this SNP increases breast cancer susceptibility [32].

Correlation between expression level, subcellular localization, and prognosis and treatment response varies between cancer types and even within same cancer type depending on the tumor characteristics [69]. For instance, a study conducted on tissue microarrays (TMAs) from gastro-esophageal cancer, ovarian cancer, and pancreatico-biliary cancer has shown APE1 overexpression in these tumors by IHC analysis. In pancreatico-biliary cancer, the absence of cytoplasmic APE1 in tumors with nuclear APE1 expression was correlated with a poor tumor differentiation, greater stage, and vascular invasion [29]. Interestingly, elevated serum APE1 concentration, as determined by ELISA, has been associated with lymph node metastasis in gastric cancer patients [30].

Furthermore, the role of APE1 as a potential early diagnostic biomarker for prostate cancer was concluded in a study that demonstrated, with IHC, an increase in both nuclear and cytoplasmic APE1 expression in prostate cancer and prostatic intraepithelial neoplasia (PIN) compared with benign hypertrophy (BPH) [33]. Moreover, upregulation of APE1 has been observed, using RT-qPCR, in human hepatocellular carcinoma (HCC) tissues compared with cirrhotic liver tissues [35]. In line with these findings, the value of APE1 as an early diagnostic biomarker for HCC has been confirmed in a recent study by measuring serum APE1 levels. Analysis using ELISA revealed a significantly higher concentration in serum samples enriched from patients with HCC compared with samples from individuals with cirrhotic and normal livers [34]. Similarly, serum APE1 has been suggested as a promising bladder cancer diagnostic biomarker as higher concentrations determined by ELISA have been found in samples from cancer patients compared with those retrieved from healthy individuals [36].

Interestingly, colorectal cancer stem cells exhibited higher expression of APE1, as determined by qPCR compared with non-stem cells from the same tumor specimen [37]. APE1 Asp148Glu and hOGG1 Ser326Cys polymorphisms, investigated by PCR and restriction fragment length polymorphism (RFLP) have been associated with increased risk for the same cancer type as higher frequency of the polymorphisms has been detected in blood samples enriched from CRC patients compared with healthy subjects [38]. Furthermore, IHC analysis of APE1 expression in 95 head and neck cancer tumors treated with chemotherapy and radiotherapy showed a correlation between loss of nuclear APE1 expression and better prognosis and treatment response [39]. Furthermore, a high expression level of APE1 mRNA in human melanoma tumors was correlated with poor survival [40].

### XPF/XPG

The heterodimer endonuclease complex “excision repair cross-complementing group 1 xeroderma pigmentosum complementation group F” (ERCC1-XPF) and the endonuclease “xeroderma pigmentosum complementation group G” (XPG) serve as substantial endonuclease activity drivers for nucleotide excision repair (NER) in both sub-pathways; global genome (GG) and transcription-coupled (TC). Although XPF is the only component that accounts for the nuclease activity of the heterodimer ERCC1-XPF, ERCC1 is essential for the function of the complex as it regulates localization and binding to DNA in addition to activation of other proteins involved in NER. Upon recognition of DNA damage caused by bulky adducts, ERCC1 protein is heterodimerized with XPF to form the heterodimer ERCC1-XPF that cleaves in the 5′ direction upstream the damage following XPG incision at the 3′ end downstream the damage. After a dual incision is performed, other NER proteins proceed with the DNA repair. ERCC1-XPF is also recruited in other types of DNA repair machinery inclusive inter-strand crosslink (ISC) and double-strand break (DSB) repair, rendering this protein into a key element for the repair of DNA damage caused by chemotherapeutics, specifically platinum-based agents [21]. As such, it was tempting to explore the potential of ERCC1-XPF as a prognostic and predictive biomarker for platinum-based chemotherapy. ERCC1 overexpression has been associated with poor response to platinum chemotherapy based on research on lung, cervical and ovarian cell lines [21]. ERCC1 predictive role has also been evaluated by analyzing tumor samples, demonstrating increased treatment response to cisplatin in correlation with negative staining in non-small cell lung cancer [42]. Consistently, as minimum as two cisplatin treatments were sufficient to cure xenografts with deficient ERCC1, whereas ERCC1-proficient melanoma xenografts were resistant to the treatment [71]. However, in gastric tumors elevated levels of the protein, as investigated by IHC, indicated better treatment response and enhanced overall survival [44]. Additionally, improved overall survival for colorectal cancer patients has been correlated with low ERCC1-mRNA expression in tumors investigated by RT-qPCR [45]. Furthermore, high expression of XPG at the RNA level in ovarian cancer has been associated with poor overall survival compared with patients with low expression [41], while XPF and XPG knockdown in osteosarcoma cell line resulted in better platinum-based treatment response [46]. Furthermore, IHC analysis revealed elevated expression of XPG in gastric tumors compared with benign lesions, indicating its involvement in malignancy progression and suggesting the utilization of XPG as a promising diagnostic biomarker [21]. Conclusively, the components of this endonuclease complex differ in predictive, prognostic, and diagnostic value based on the cancer type.

### MRN complex

The MRE11-RAD50-NBS1 (MRN) is a major component in homologous recombination (HR) and non-homologous end-joining (NHEJ) pathways for DSBs repair [21, 22]. The MRN complex degrades the 3′ single-strand DNA overhang yielded by resection of double-stranded DNA, promoting the progression of DNA synthesis [21]. Loss of expression of any of the proteins may abrogate the activity of the complex. Loss of MRN complex expression has been reported in several cancer types including gastric, endometrial, breast, colorectal, and bladder cancer [21]. In breast cancer, immunohistochemical analysis showed decreased expression of each of the three MRN proteins in breast cancer tissues compared with normal control tissues. Additionally, a correlation between the expression of the proteins to each other was found in the majority of the samples [47]. Whereas in colorectal and endometrial cancer, mutations in MRE11 resulting in reduced expression of MRE11 and impaired function of the MRN complex have been observed [48]. Interestingly, an investigation of MRN-complex expression in a cohort of 521 endometrial carcinoma and 10 cell lines revealed MRE11 protein absence in 30.7% of the tumors. Worth mentioning is that loss of MRE11 was correlated with loss of the other components of the MRN-complex [49]. MRE11 has also demonstrated high potential as a predictive biomarker for radiotherapy in bladder cancer patients. High protein expression, analyzed by IHC, has been associated with improved survival. This indicated the potential of MRE11 to improve cure rates by providing ameliorated stratification of patients into cystectomy or radiotherapy treatment [50]. Moreover, gastric carcinoma with high-level microsatellite instability is known for frequent mutations in coding and non-coding mononucleotide repeats. It has been demonstrated that mutations of the intronic poly(T)11 repeat in MRE11 are specifically associated with this phenotype, as well as with diminished MRE11 protein expression as visualized by direct immunoblotting [51].

### TREX2

Three prime repair exonuclease (TREX2) is a 3′–5′ deoxyribonuclease that is involved in DNA degradation, replication, repair, and recombination. Hence it contributes to genome editing, working on both double-stranded (dsDNA) and single-stranded DNA (ssDNA). TREX2 is an Mg^2+^-ion-dependent exonuclease of the DNAQ-like exonuclease family, and it is specifically expressed in the skin, esophagus, tongue, and forestomach. In skin, TREX2 is expressed in the cytosol but mainly accumulated in the nuclei of suprabasal keratinocytes and is regulated during keratinocytes differentiation [23].

TREX2 knockout in a mouse model has been associated with increased susceptibility to skin carcinogenesis upon exposure to ultraviolet B (UVB) radiation or topical treatment with a DNA-damaging carcinogen 7, 12-dimethylbenz [a] anthracene [52]. This finding was associated with impaired degradation and removal of damaged DNA, an important function for epidermis integrity maintenance, and decreased inflammatory response. TREX2 deletion suppressed upregulation of Interleukin 12 (IL12) and Interferon-gamma (IFNγ), the main cytokines involved in anticancer immunity and DNA repair. Moreover, TREX2 contributed to DNA repair and apoptosis in UV-treated keratinocytes. Therefore, it was concluded that TREX2 suppresses keratinocyte-driven carcinogenesis through promoting keratinocyte cell death in addition to the inflammatory and immune response that are key elements of anticancer mechanism.

Remarkably, Manils et al. demonstrated deregulated TREX2 expression in squamous carcinomas by IHC analysis of human samples of head and neck squamous cell carcinoma (HNSCC), cutaneous squamous cell carcinomas (CSCCs) and precancerous actinic keratosis lesions. TREX2 expression pattern varied with the variation of tumor differentiation and metastasis. In HNSCC and CSCCs, the absence of TREX2 expression was correlated with a more advanced and metastatic phenotype. While elevated expression was associated with well-differentiated and non-metastatic CSCCs.

Several germline SNPs were demonstrated by sequencing tumor and blood samples retrieved from HNSCC patients and healthy subjects. The single amino acid variants were all identified in male patients, and since TREX2 is located in the X-chromosome they were expressed in homozygosis. These variants were assayed for exonuclease activity using ssDNA and dsDNA. Significantly decreased relative dsDNAse activity was associated with the R156L variant, while no remarkable change in nuclease activity was reported for the remaining variants, generally indicating that these mutations do not contribute to a complete loss of function [52].

### SND1

Staphylococcal nuclease domain containing-1 (SND1) protein plays substantial intracellular roles comprising; transcriptional coactivation, mRNA splicing, and nuclease activity in RNA- interference as a component in the RNA-induced silencing complex (RISC) [72]. The multifunctionality could be explained by the structure of SND1 that includes five repeated staphylococcal nuclease homology domains suggested to be interaction location with nucleic acids, and a Tudor-homology domain for interaction with proteins [57].

SND1 involvement in the degradation of tumor suppressor mRNA as a part of RISC machinery that shows elevated activity in human hepatocellular carcinoma (HCC) cells. IHC analysis of TMAs has shown overexpression of HCC in tumors. Conclusively, it is suggested that SND1 contributes to carcinogenesis in HCC [53]. Moreover, SND1 has been linked to angiogenesis in HCC cell lines, basically through activation of angiogenic factors Angiogenin and C-X-C Motif Chemokine Ligand 16 (CXCL16) [54]. Correspondingly, IHC analysis revealed overexpression in HCC samples compared with normal liver samples [53]. Another study has suggested SND1 implication in cell motility, one of cancer cells’ hallmarks. Morphology change in HCC cell lines was observed upon manipulation of SND1 expression level. It has been demonstrated that SND1 increases angiotensin II type 1 receptor (AT1R)- mRNA stability and hence AT1R level, which in turn leads to activation of transforming growth factor beta (TGFβ) downstream cascade. And since TGFβ is known as a key driver of cell invasion through epithelial-mesenchymal transition (EMT), elucidation of SND1 activation of this pathway provides an explanation for the role of SND1 in cell motility increase. Correspondingly, IHC staining of HCC samples showed remarkably elevated SND1 and AT1R expression compared with matched normal tissue samples, suggesting its role as a diagnostic marker and confirming the notion of correlation with AT1R increase [72].

IHC analysis of SND1 in human prostate cancer tumors showed the protein expression, mainly localized in tumor cells cytoplasm in the cancer specimen, compared with low or negative SND1 expression in hyperplastic and normal samples. The staining intensity was positively correlated with tumor grade and aggressiveness. In-situ hybridization of randomly selected samples from the IHC-analyzed cohort showed consistently elevated SND1-mRNA in cancer cells and low or negative expression in the normal counterparts. Moreover, SND1-knockdown in the prostate cancer cell line PC3, using Small interfering RNA (siRNA), was correlated with diminished cell growth. Conclusively, SND1 has been suggested as a promising diagnostic biomarker in prostate cancer [56].

In a study by Tsuchiya et al. upregulation of SND1-mRNA was reported to be upregulated in human colon cancer tissues inclusive in the early-stage lesions, as well as in colon cancer cell line IEC6. Consistently, SND1 overexpression was visualized by IHC in chemically induced colon cancer tumors in a rat model but also in precancerous lesions. Previous data suggest early upregulation of SND1 in colon cancer and its potential contribution to early colon carcinogenesis, mainly as a key regulator of colon cancer-development mediators such as β-catenin and APC [57].

Using microarray analysis of RNA retrieved from wild type (WT) and SND1-knockdown breast cancer SCP28 cell lines, it was shown that SND1-knockdown is strongly correlated with downregulation of a panel of oncogenes and metastatic genes such as Angiopoietin-like 4 (ANGPTL4), Epiregulin (EREG) and Inhibitor of DNA Binding 1 (ID1) known for being implicated in chemoresistance. Thus, SND1 was suggested as a mediator of oncogenic, metastatic, and antiapoptotic genes expression signatures. Data from microarray analyses of SND1 expression of primary breast cancer samples cohort have correlated high SND1expression with reduced metastasis-free survival especially lung metastasis. In line with this finding, SND1 was found to contribute to lung metastasis in rat experimental models [55]. Thus, accumulative data indicate the role of SND1 as a diagnostic but also a prognostic cancer biomarker.

### DNaseI

Deoxyribonuclease I (DNaseI) is an extracellularly active endonuclease that cleaves ssDNA or dsDNA in a Ca^2+/^Mg^2+^ dependent manner, resulting in 5′-phosphoryl dinucleotides and 5′-phosphoryl-oligonucleotides [58, 73]. DNaseI protein exhibit polymorphism governed by 6 alleles yielding in 3 common and 7 rare phenotypes in the Japanese population [74]. The enzymatic activity of DNaseI is distributed specifically in the kidney, liver, urine, pancreas, semen, and digestive tract [58]. In addition, it is present in blood where it contributes to the degradation of circulating serum DNA [75]. Besides being the main responsible for serum nucleolytic activity, DNaseI is involved in apoptotic DNA fragmentation [73].

The role of DNaseI as a cancer biomarker was investigated by Tsutsumi et al. already in the late nineties. The research group demonstrated a remarkable association between the high frequency of DNaseI phenotype2 and gastric carcinoma. While no difference in polymorphism distribution was found in patients with benign gastric conditions compared with the healthy subjects. Phenotyping was conducted on urine samples from the participants using electrophoresis in thin polyacrylamide gel followed by immunoblotting with an antihuman DNaseI antibody [58]. Thus, DNaseI phenotype2 has been suggested as a biomarker that identifies individuals with a risk to develop gastric carcinoma [58]. Similarly, the phenotype2 of the endonuclease has been suggested as a promising biomarker for identification of patients with a high risk of or harboring colorectal carcinoma as high frequency of phenotype2 of has been significantly correlated with colorectal carcinoma. Polymorphism distribution, however, has not shown a significant difference between benign disease and control arms [59]. Furthermore, no significant phenotype distribution has been found in lung cancer, indicating that DNaseI is not a lung cancer susceptibility gene [74].

### RNaseL

Ribonuclease L (RNaseL) is an abundantly expressed endonuclease in most of the bodily tissues. The first uncovered function of this nuclease is its vital role in innate immunity against viral infection, mainly due to being a key element in the 2′-5′-oligoadenylate synthetase (OAS)/RNaseL pathway. Upon recognition of the pathogen dsRNA, IFN inducible OAS is activated to produce 2′-5′-oligoadenylates (2-5A) from adenosine triphosphate (ATP). RNaseL is expressed in a latent form that is activated by 2-5A oligomers to produce the catalytically active form. The catalytically activated enzyme hydrolyzes both viral and cellular ssRNA, inhibiting the viral infection. Namely, RNaseL cleaves at the 3′ sides of UpAp and UpUp dinucleotides [76, 77]. The RNA-cleavage products induce IFN-β production through activation of retinoic acid-inducible-I-like receptors [77]. Its role in innate immunity exceeds the antiviral function to providing protection to the central nervous system against virally induced demyelination. Moreover, it has been suggested that RNaseL is implicated in blocking bacterial infections [77], mainly through promoting proinflammatory cytokine induction and regulating endosomal pathways that eliminate bacteria [78]. Besides, it has been suggested that RNaseL is a key factor in the host immunity against cancer. An additional role is an involvement in adipogenesis [79].

Research has unraveled a correlation between *RNaseL* mutations and prostate cancer, leading to the classification of *RNaseL* gene as a prostate cancer susceptibility gene [60]. *RNaseL* location within the hereditary prostate cancer 1 (HPC1) region at 1q25.3 indicates a role as a tumor suppressor in a direct or indirect manner during the malignant transformation of prostate cancer [61]. Not surprisingly, a correlation has been demonstrated between several SNPs of the human *RNaseL* and hereditary and sporadic prostate cancer. However, the mechanism behind this correlation remains to be fully deciphered [60]. Interestingly, the RNase LR462Q variant was correlated with reduced RNaseL activity, as measured by comparing intact 28S and 18S rRNA with specific rRNA cleavage products on RNA chips. The deficient activity was attributed to decreased dimerization of the enzyme to its active form. The prostate cancer risk associated with this variant was suggested to be correlated with enzymatic deficiency, which in its turn contributed to decreased apoptosis inducement [80]. While several studies have demonstrated a correlation of germline *RNaseL* mutations with prostate cancer, the presence of somatic mutations that inactivate *RNaseL* gene is rare in sporadic prostate cancer according to a mutational analysis of prostate cancer specimen and cell lines [62].

Due to its role in the antiviral machinery, a correlation between RNaseL dysregulation and virally induced cancers was hypothesized. As such, analysis of tumor DNA in the uterine cervix, HNSCC, and breast cancer samples, and the patients’ somatic tissue genotyping were performed. Results demonstrated a correlation between increased cancer risk and RNaseL SNP rs3738579 in these cancer types, suggesting that *RNaseL* is not limited to prostate cancer but rather is a general cancer susceptibility gene. Indeed, a chromosomal gain of the HPC1 region, where *RNaseL* is located, has been frequently correlated with uterine cervix cancer and HNSCC. In addition, amplification of the chromosome arm 1q in total was suggested to be an early step in breast carcinogenesis [63].

### RNaseI

Ribonuclease I (RNaseI) is the most well-known member of the RNaseA superfamily of nucleases and has a wide utility in microbiology laboratories as a commercially available RNA degrading reagent. From a physiological standpoint, RNaseI belongs to the pancreatic-type secretory nucleases, a subclass of the RNaseA superfamily which is involved in host immunity against cancer [81, 82]. Like other RNases of this family, RNaseI is inhibited by the cytosolic protein mammalian ribonuclease inhibitor (RI). RI binds to RNase with a high affinity leading to the inactivation of the ribonuclease’s catalytic activity [83]. The RNaseI endonuclease degrades RNA at the 3′-end of pyrimidine bases, with a preference to dsRNA over ssRNA and poly(C) as a substrate [82]. N-glycans of RNaseI purified from pancreatic cells of healthy donors were compared with their counterparts from media conditioned with Capan-1 or MDAPanc-3 pancreatic adenocarcinoma cell lines. The study revealed different glycosylation trends manifested by the difference between the characterized glycans. This led to the idea of employing RNaseI as a potential diagnostic biomarker for pancreatic adenocarcinoma, namely through availing the difference in glycosylation patterns. Interestingly, glycans produced from different sources exhibited distinct epitopes that enabled researchers to identify antibodies that specifically react with RNaseI secreted by healthy cells or cancer cells using several immunoassays. Thus, serum RNaseI was indicated as a promising diagnostic biomarker for pancreatic adenocarcinoma that is challenging to diagnose due to the absence of distinct symptoms in the clinical picture [64]. Later, researchers improved this notion by specifically targeting the difference in glycosylation status of Asparagine (Asn) residue of an RNaseI glycoprotein. Antibodies that specifically bind to unglycosylated Asn^88^ were developed and utilized in differential immunoassays and WB. It was thus demonstrated that levels of serum RNaseI containing N-glycosylated Asn^88^ are elevated in samples obtained from pancreatic cancer patients compared with those from healthy participants [65].

### Serum RNase activity

It has been well established that RNases participate in cancer growth control, for instance, some of the secretory RNaseA family has been demonstrated to exhibit a tumor-suppressive role [81]. Serum RNase activity was investigated on the background of reported serum nucleolytic activity in association with cancer, including pancreatic cancer, and in acute necrotizing pancreatitis. Serum RNase enzymatic activity was assayed using two substrates: t-RNA (T) from *E. coli* MRE 600 and the synthetic polycytidylic acid (poly-C) at pH of 7.4 and 6.6, respectively. Elevated serum ribonuclease activity (SRA) was expressed in terms of the amount of bovine serum RNaseA from the bovine pancreas in ng/ml that yields the same extinction coefficient at 260 nm wavelength. Elevated SRA was found in samples from patients with pancreatic carcinoma compared with healthy individuals. Increased activity was observed in presence of impaired renal function as well as in patients with chronic pancreatitis, but discrimination between the latter and carcinoma samples was achieved. Although carcinoma samples exhibited significantly higher activity compared to chronic pancreatitis samples, the sensitivity was pretty low. Besides, a remarkable overlap between pancreatic carcinoma and normal samples from age-matched controls was observed, concluding that serum RNase was not an optimal biomarker for pancreatic carcinoma detection. Variation in the molecular weight and multiple sources of serum RNase were suggested as explanations for the low diagnostic value of serum RNase using this method [66].

### Nuclease activity as a diagnostic biomarker using nucleic acid probes as substrates

Nucleases have frequently been reported for their diagnostic value in cancer, by studies conducted on human tissue specimens as well as cell lines. As previously discussed, research has mainly focused on nucleases as proteins, investigating the difference in expression at the RNA level or being targeted with antibodies as a potential diagnostic biomarker. However, just a few studies attempted to take advantage of nucleases’ dynamic catalytic activity as a biomarker. And when available, such studies concerned one nuclease at a time discarding the importance of a global nuclease activity as a discriminative feature between cancer and healthy. Indeed, the latest technological advancements have led to the establishment of various diagnostic methods that form the diagnostic landscape we currently recognize, building on incremental knowledge rather than introducing a breakthrough. In this regard, nuclease activity has remained a potential but not yet fully explored biomarker mostly due to the lack of robust tools and standardized protocols to allow specific and sensitive measurement of nuclease activity correlated with cancer, in a reproducible and reliable manner.

Since nucleases have been previously found to be overexpressed in cancer, at protein and RNA levels, it was postulated that nuclease activity could be also elevated in cancer compared with a normal condition. Hernandez et al. have demonstrated this concept by developing a library of nucleic acid probes containing chemically modified nucleosides, as substrates for breast cancer-associated nuclease activity [84]. The probes have been designed and manufactured as described in [85] to contain chemical modifications at specific nucleosides, rendering the sequences resistant to the bodily nucleases while prone to cleavage by cancer associated nucleases [84]. The probes were synthesized with a fluorophore (fluorescein amidite, FAM) and a quencher (tide quencher 2, TQ2) at the 5′ and 3′ ends respectively, rendering the probes in an initial off state due to their proximity. Upon nuclease cleavage, this proximity is disrupted leading to an “on” state when the fluorophore recovers its properties and emits a fluorescent signal that can be quantified using a plate reader. Utilizing a library of these probes, it was possible to identify breast cancer cell lines from healthy fibroblasts by assaying cell surface-associated nuclease activity. SKBR3 breast cancer cells exhibited significantly higher nuclease activity, as reported by fluorescence intensity, specifically towards 2´-O-methyl purine-modified sequences compared with the healthy counterparts [84]. These results were intriguing and warranted further investigation by improving the design and incorporating nucleic acid probes as detection tools in strategies that employ nuclease activity as a biomarker **(**Fig. 1**)**. More specifically, readily available chemically modified nucleosides enable the design and development of more tailored oligonucleotide libraries with enhanced sensitivity and specificity towards the target nuclease. The concept of screening and probe design in an iterative manner to yield best performing probes has been comprehensively described in [86]. Worth mentioning is that nuclease activity has been exploited as a biomarker for the detection of cancer cells by an independent research group, where researchers have been able to detect circulating tumor cells enriched from breast cancer patients based on the cells’ nuclease activity [87].

Very promising results were achieved in a study that comprised paired breast samples (cancer and healthy), where a panel of 3 chemically modified nucleic acid probes could successfully differentiate between cancer and healthy samples. Through harnessing cancer-associated nuclease activity towards the probes, discrimination between cancer and healthy was achieved with high accuracy, sensitivity, and specificity [88]. This highlights the great potential of nuclease activity as a functional diagnostic biomarker but also as a complementary diagnostic strategy along with the golden standard of histopathological diagnosis. In another unpublished study, HNSCC cells have been differentiated from healthy fibroblasts relying on respective associated nuclease activity profile towards chemically modified oligonucleotides. Cancer-associated nuclease activity towards specific nucleic acid probes was higher compared with the healthy cells.

Altogether, data point to weigh of implementing nuclease activity as a novel cancer diagnostic biomarker. The power of this method lies in being responsive to the demands in diagnostics through a flexible probe design that is adaptive to cancer type and sample, and can be integrated into an imaging modality and detects cancer in a specific and sensitive manner.

### Advantages, potential and future directions of nuclease activity as a biomarker in Cancer diagnosis

Cancer is one of the leading causes of death worldwide, which poses serious challenges to health, wellbeing, and financial domains [89]. An efficient early diagnosis is well recognized to be an essential factor of successful treatment and hence an improved survival and quality of life. Although much has been achieved in the diagnostic field in the latest decades, there is still an immense need for an improvement towards an earlier diagnosis. More efficient and accurate biomarkers are also a need as, to say the least, both diagnostic systems and biomarkers are not problem-free. Most of the biomarkers do not exhibit sufficient sensitivity and/or specificity as single entities. And diagnostic systems suffer from a low analytical sensitivity range of detection, imperfect reproducibility, practical complexity in the application and results analysis and interpretation, in addition to high cost and invasiveness [2].

While most of the studies focus on comparing the expression status of a specific nuclease, at protein or mRNA level, for a cancer type, less effort has been dedicated to interrogating the dynamic nucleolytic activity of these enzymes as a potential cancer biomarker. We and others have been among the leading research groups that properly discussed and developed this notion [84, 86, 87]. In fact, the reported difference in nuclease profile as protein or mRNA between cancer and normal has led Hernandez et al. to anticipate that nuclease activity signature could be recruited as a diagnostic biomarker taking advantage of the dynamic property of these enzymes. Nuclease activity profile has been successfully utilized to differentiate between breast cancer cells and healthy counterparts [84], HNSCC cells from the normal cells (Hernandez Lab, unpublish data), and breast cancer tissue samples from benign samples with high accuracy [88]. This reflects the utility of nuclease activity assaying as a diagnostic approach but also underlines the diversity of nucleases where nucleic acid probes can be tailored to specifically detect each cancer type.

Implementation of nuclease activity as a biomarker tackles the accuracy problem through developing nucleic acid probes with enhanced sensitivity and specificity in an iterative manner [86]. In that, the probes’ design is improved based on the sequence of candidate oligonucleotides from each screening round. Modulable screening for nuclease activity associated with a specimen, flexibility in probe design, and diversity of nucleases allow precision. Thus, generating a probe with desirable sensitivity and specificity is achieved. In fact, optimization enables to design a specific probe for each cancer type [86].

Using nuclease activity scores an advantage over conventional methods, such as ELISA, because signal amplification is an innate feature for nucleases. The interaction between nuclease and probes is dynamic in nature and allows degradation of several probes by the same nuclease/s, providing as low detection limit as picomoles or femtomoles. A clear advantage is to eliminate dependence on tumor size when imaging modality is involved.

A great potential of nuclease activity as a biomarker lies in the ability of its integration in imaging modalities, using the catalytic properties of nucleases towards nucleic acid probes that work as biorecognition molecules. When incorporated in an imaging technique with a high resolution such as magnetic resonance imaging (MRI), the result is an activatable probe that enables signal detection upon degradation by cancer nucleases. MRI-probes that are specifically degraded by micrococcal nuclease associated with *Staphylococcus aureus* have been designed and tested in-vitro with very promising results [90, 91]. The future direction is to apply the same principle in cancer detection. An obvious advantage is the elimination of need for biopsy to confirm the diagnosis, besides the activatable probes’ unique features of high spatial resolution, sensitivity, and specificity through augmentation of target-to-background ratio [91].

Another issue in current diagnostic strategies is the analysis of the same molecule at different levels; DNA, mRNA, and protein by different assays. The latter is assayed with antibodies that vary in source and sensitivity, confounded with inter-batch differences in used kits. Choice of the method is mainly governed by a tradeoff between sensitivity and cost-effectiveness [2]. In comparison, nuclease activity assay offers a single platform for detection with no batch-to-batch differences in probes, which provides high reproducibility and eliminates the ambiguity in analysis and interpretation of results. It is worth mentioning that manufacturing nucleic acid probes overcomes the time-intensive and cumbersome production of antibodies widely used in clinical immunoassays.

The applicability extends from the promising results in vitro, ex-vivo using tissue samples and in-vivo in animal models [85], besides facile adaption in imaging modalities. This renders nuclease activity assaying into a “wide spectrum” method that targets the most significant problems in conventional diagnostics.

## Conclusion

To sum up, early cancer detection is a key factor in disease management by providing treatment at an early stage, optimizing therapeutic choice, and improving survival. Currently available diagnostic strategies still confront obstacles resembled by lack of accuracy and therefore novel biomarkers with enhanced sensitivity and specificity, in addition to improved imaging modalities, are pivotal elements to solve the problem. Nucleases are among the numerous molecules that have garnered the research interest as diagnostic biomarkers using the available analytical platforms. We and others have demonstrated the successful use of cancer-associated nuclease activity’s blueprint as a means of differentiation between cancer and healthy cells. As such, we have provided evidence for the utility of nuclease activity as a potential diagnostic biomarker that could be implemented in clinical use. Thus, using nuclease activity opens the window for possibilities beyond the currently available cancer biomarkers. But most importantly, it lays down a principle of developing methods based on nuclease activity as a biomarker.

## Data Availability

Not applicable.

## Abbreviations

AP: apurinic/apyrimidinic
APC: adenomatous polyposis coli
APE1: Human apurinic / apyrimidinic endonuclease1
Asn: Asparagine
ATP: adenosine triphosphate
AT1R: angiotensin II type 1 receptor
BER: base excision repair
BPH: benign hypertrophy
CpG: cytosine-phosphate-guanine
cSCCs: cutaneous squamous cell carcinomas
CXCL16: C-X-C Motif Chemokine Ligand 16
DNaseI: Deoxyribonuclease I
DSB: double-strand break
dsDNA: double stranded
ELISA: enzyme-linked immunosorbent assay
EMT: epithelial–mesenchymal transition
EREG: Epiregulin
ERCC1-XPF: The heterodimer endonuclease complex “excision repair cross complementing group 1 xeroderma pigmentosum complementation group F”
FAM: fluorescein amidite
FEN1: Flap endonuclease1
HCC: human hepatocellular carcinoma
HNSCC: head and neck squamous cell carcinoma
HPC1: hereditary prostate cancer 1.
HR: homologous recombination
IFNγ: Interferon gamma
IHC: immunohistochemistry
IL12: Interleukin 12
ISC: inter-strand crosslink
MRI: magnetic resonance imaging
MRN: The MRE11-RAD50-NBS1
NER: nucleotide excision repair
NHEJ: non-homologous end joining
OAS: the 2′-5′-oligoadenylate synthetase
PBMC: peripheral blood mononuclear cells
PCR: polymerase chain reaction
PIN: prostatic intraepithelial neoplasia
RFLP: restriction fragment length polymorphism
RI: ribonuclease inhibitor
RISC: RNA-induced silencing complex
RNaseI: Ribonuclease I
RNaseL: Ribonuclease L
RT-qPCR: quantitative real-time polymerase chain reaction
SND1: Staphylococcal nuclease domain containing-1
SNP: nucleotide polymorphism
siRNA: Small interfering RNA
SRA: serum ribonuclease activity
ssDNA: single stranded DNA
TCGA: Cancer Genome Atlas
TGFβ: transforming growth factor beta
TMAs: tissue microarrays
TQ2: tide quencher 2
TREX2: Three prime repair exonuclease
UVB: ultraviolet B
WB: Western Blot
WT: wild type
XPG: the endonuclease “xeroderma pigmentosum complementation group G”
2-5A: 2′-5′-oligoadenylates
